# Evaluation of suitable reference genes for normalization of real-time reverse transcription PCR analysis in colon cancer

**DOI:** 10.1186/1756-9966-29-144

**Published:** 2010-11-08

**Authors:** Lise Aa Sørby, Solveig N Andersen, Ida RK Bukholm, Morten B Jacobsen

**Affiliations:** 1 Quality and Research Department, Ostfold Hospital Trust, 1603 Fredrikstad, Norway; 2 Dep. of Pathology, Akershus University Hospital, 1478 Lørenskog, Norway; 3 Institute of Clinical Medicine, University of Oslo, 0316 Oslo, Norway; 4 Dep. of Surgery, Akershus University Hospital, Lørenskog, Norway; 5 Dep. of Health Promotion, Akershus University Hospital, Lørenskog, Norway; 6 Dep. of Chemistry, Biotechnology and Food Science, Norwegian University of Life Sciences, 1432 Ås, Norway

## Abstract

**Background:**

Real-time reverse transcription PCR (qRT-PCR) is frequently used for gene expression quantification due to its methodological reproducibility and sensitivity. The gene expression is quantified by normalization to one or more reference genes which are presumed stably expressed throughout a given experiment. The aim of this study was to validate a standardized experimental setup to identifying reference genes for normalization of qRT-PCR in the metastatic and non-metastatic colon cancer.

**Methods:**

In this study, expression of 16 commonly used reference genes was quantified in tumour tissue and individual-matched normal mucosa in 18 non-metastatic colon cancer patients and 20 colon cancer patients with distant metastases using TaqMan Low Density Array (TLDA). The expression stability was determined and compared by means of geNorm and NormFinder.

**Results:**

Two pairs of genes, HPRT1/PPIA and IPO8/PPIA, were identified to be suitable to normalize gene expression data in metastatic and non-metastatic colon cancer patients, according to geNorm and NormFinder respectively.

**Conclusion:**

We propose a standardized approach of finding the most suitable reference gene(s) in every qRT-PCR experiment using TLDA.

## Background

qRT-PCR is one of the most sensitive methods for mRNA detection and quantification. The method has also become the preferred method for validating results obtained by other techniques, such as microarray [1]. There are differences among different qRT-PCR assays due to biological and technical variations [2,3]. In order to identify truly gene specific variations it is important to use a suitable normalization method. One of the most commonly used approaches involves relative quantification of target genes against one or more reference genes which are thought to be stably expressed in the examined tissue [4]. There have been a number of reports that demonstrate that the expression levels of putative reference genes vary extensively in different tissues and diseases and thus are unsuitable for normalization purposes [5-15]. Consequently, each research group has to validate multiple reference genes in their own experimental setup and normalize qRT-PCR data against a few reference genes tested from independent pathways using at least one algorithm. It appears that improvements in methods of identifying reference genes are more important than the identification of the particular reference genes themselves [16].

It has been argued for use of multiple genes in the normalization of qRT-PCR analysis and several algorithms have been developed [17-20]. Vandesompele et al., 2002, used the geometric mean of the most stable genes to improve the accuracy of the analysis in a method called geNorm [19]. This method relies on the principle that the expression ratio of two ideal reference genes is identical in all samples regardless of the experimental conditions. For every reference gene geNorm determine the pairwise variation with all other reference genes. The average pairwise variation of a particular gene is defined as the internal control stability measure; M. Genes with the lowest M values are the most stable ones. Another algorithm in which the expressional stability of genes is evaluated is NormFinder [17]. NormFinder estimates the intra-group and the inter-group expression variation. Both of these sources of variation are combined into a stability value. This method can account for heterogeneity of the tested tissue samples. Genes with the lowest stability value have the most stable expression.

Colorectal cancer is among the most frequent malignant diseases worldwide, and is one of the leading causes of cancer-related deaths [21]. The majority of colorectal tumours develop along a well-defined adenoma-carcinoma sequence in which oncogenes are activated and tumour suppressor genes lose their function [22]. Despite a high 5-year survival rate in early colorectal cancer, only 10% of the patients with distant metastases survive after five years [23]. Thus, it is important to elucidate the biology that contributes to this progression, especially those processes that facilitates the switch to invasive and metastatic disease. Biological changes are a result of partly differential gene expression, which can be confirmed by qRT-PCR. It is necessary to validate reference genes in the particular experimental system in order to trust the differential gene expressions which are detected. Previous studies have tried to find universally stable reference genes across several types of cancers, including colon cancer [24-26]. Recent reports, however, claim that stably expressed genes in one tumour type may not predict stable expression in another tumour type [12,27]. Moreover, results in one tumour type, like colorectal cancer, show stably expressed genes in one experimental in which are different from the stably expressed genes in another experimental setup [28-30]. Hence, reference genes should be validated and selected in every experiment in any tissue type. Recently, it has been suggested that the focus should be on introducing and validating novel approach for reference gene identification and standardizing experimental setup rather than giving general suggestions for different tissues [16]. Applying TaqMan Low Density Array (TLDA) to examining reference genes is a step towards a more standardized experimental setup. TLDA was evaluated in colorectal cancer by Lü et al., 2008, as a roughly robust and labour-saving method for gene quantification compared with routine qRT-PCR [31]. Well-designed TaqMan probes require little optimization, and TLDA allows simultaneously real-time detection of many gene products in several samples offering higher through put than established single array method [31,32]. Hence, in the present study we used TLDA to find potential reference genes for data normalization in qRT-PCR experiments in metastatic and non-metastatic colon cancer patients. The gene expression of 16 commonly used reference genes in tumour tissue and individual-matched normal mucosa of metastatic and non-metastatic colon cancer patients were analyzed and the expression stability was determined and compared using geNorm and NormFinder.

## Methods

### Patients and tissue specimens

RNAlater-stored tumour tissue samples and individual-matched normal mucosa were obtained from 38 patients with colonic adenocarcinoma who underwent resection at Akershus University Hospital Trust between 2004 and 2009. The dissected tissue samples were collected in the operating room and stored immediately in approximately five volumes of RNAlater (Ambion Inc., Austin TX, USA) and frozen at -80°C. Eighteen patients with non-metastatic disease, Dukes B (with a minimum of 12 negative lymph nodes) where no metastases occurred during 5 years follow up, and 20 patients originally staged as Duke C who displayed distant metastases during a 5 year follow-up (Duke C) or patients classified as Dukes D were included in the study. There were 22 women and 16 men with a mean age of 69 +/- 14 years (range 29-92) at surgery. Three sectioned pieces of the tumour samples were made. The central piece was further processed for RNA isolation, while the two end pieces were fixed in formalin and embedded in paraffin (FFPE). Four μm sections of FFPE samples were stained with Hagens Hematoxylin and examined by a pathologist for determination of percentage tumour cells. To avoid bias from necrosis or minimal tumour representation we included tumour tissue samples with more than 70% tumour cells.

### mRNA isolation

Total RNA isolation was performed using the method of Wei and Khan, 2002, [33] modified according to T. Lüders (unpublished work) to also include miRNA for further analyses. Approximately 60 mg frozen tissue was homogenized in TriReagent (Ambion) using Mixer Mill MM301 (Retch) for 2 × 2 min at 30 Hz. After phase-separation with chloroform, the aqueous phase (containing RNA) was mixed with 1.5 volumes 100% ethanol and transferred to an RNeasy Mini spin column (Qiagen). Further processing was performed following the manufacturer's protocol. A DNase treatment was included in the procedure. RNA was eluted in 60 μl RNase-free water and stored at -80°C. The concentration of each RNA sample was obtained from A_260 _measurements using the NanoDrop 2000 (Thermo Fischer Scientific Inc.). The RNA integrity number (RIN) was tested by using the Agilent 2100 Bioanalyzer (Agilent Technologies).

### cDNA synthesis

Complementary DNAs (cDNAs) were produced from 1 μg RNA of each sample using the High Capacity RNA-to-cDNA Master Mix (Applied Biosystems) according to the manufacturer's instructions. The following thermal cycler conditions were used: 5 min at 25°C, 30 min at 42°C and 5 min at 85°C. Three random RNA samples were additionally run in the absence of reverse transcriptase enzyme to assess the degree of contaminating genomic DNA. Real-time PCR with genomic DNA specific assay revealed that RNA was free of genomic DNA (data not shown).

### TLDA design and preparation

TaqMan Endogenous Control Assays (Applied Biosystems) are 384-well microfluidic cards containing 16 preoptimized human TaqMan Gene Expression Assays commonly used as endogenous controls and genes that exhibit minimal differential expression across different tissues (Table 1). The assay was performed in triplicates. 50 μl cDNA (1 μg mRNA) was used as a template. Matched samples from 4 patients where loaded on each card. NTC (no template control) was added in one loading port. PCR amplification was performed using the ABI Prism 7900 HT Real Time PCR System (Perkin-Elmer Applied Biosystems, Foster City, California, USA). Thermal cycling conditions were used as follows: 2 min at 50°C, 10 min at 94.5°C, 30 sec at 97°C, and 1 min at 59.7°C for 40 cycles.

**Table 1:** Candidate reference genes included in the TaqMan Endogenous Control Assay.

Gene name	Gene symbol	Assay ID	Size (bp)	Function
18S Ribosomal RNA	18S	Hs99999901_s1	187	Part of a ribosomal subunit
Phosphoglycerate kinase-1	PGK1	Hs99999906_m1	75	Key enzyme in glycolysis
Β-Actin	ACTB	Hs99999903_m1	171	Cytoskeletal structural protein
Polymerase (RNA) II polypeptide A	POLR2A	Hs00172187_m1	61	Catalyzes the RNA synthesis from DNA
Beta-2-microglobulin	B2M	Hs99999907_m1	75	Beta-chain of major histocompatibility complex class I molecules
Peptidyl-prolyl isomerase/cyclophilin A	PPIA	Hs99999904_m1	98	Catalyzes the cis-trans isomerization of proline imidic peptide bonds in oligopeptides, accelerating folding
Glyceraldehyd-3-phosphate dehydrogenase	GAPDH	Hs99999905_m1	122	Dehydrogenase, oxidoreductase in glycolysis and gluconeogenesis
Acidic ribosomal phosphoprotein P0	RPLP0	Hs99999902_m1	105	Ribosome biogenesis and assembly
Β-Glucuronidase	GUSB	Hs99999908_m1	81	Glycoprotein, degradation of dermatan and keratin sulfates
Transcription factor IID, TATA box binding protein	TBP	Hs99999910_m1	127	TATA binding protein, general RNA polymerase II transcription factor
Hydromethylbilane synthase	HMBS	Hs00609297_m1	64	Heme synthesis, porphyrin metabolism
Transferrin receptor (p90, CD71)	TFRC	Hs99999911_m1	105	Cellular uptake of iron
Hypoxanthine-phosphoribosyl-transferase 1	HPRT1	Hs99999909_m1	100	Glycosyltransferase, purine synthesis in salvage pathway
Ubiquitin C	UBC	Hs00824723_m1	71	Protein degradation
Importin 8	IPO8	Hs00183533_m1	71	Function in nuclear protein import
Tyrosine 3 monooxygenase activation protein, zeta polypeptide	YWHAZ	Hs00237047_m1	70	Signal transduction by binding to phosphorylated serine residues on a variety of signalling molecules

### TLDA analysis and Statistical analysis

RealTime Statminer 3.0 Software (Integromics, Madrid, Spain) was used for implementation of quality controls in addition to calculation of optimal endogenous controls. This program uses the comparative Ct method for relative quantification analysis, and the results are expressed as a fold change of expression levels (DDCt values). The mean value of triplicates was applied for all calculations. Medians were used to replace missing values that occurred due to inconsistencies between replicates rather than from low expression. The detectability threshold was set to 36, meaning failing detectors are those with a Ct greater than or equal to 36. To measure the expressional stability of the candidate endogenous control genes, two commonly used programs were employed: geNorm [19] and NormFinder [17], both of which available in RealTime Statminer. Ct coefficients of variations (CtCV%) were calculated for every reference gene across all samples. All data are expressed as means ± SD. Except from the analyses in RealTime Statminer, all other calculations were performed using SPSS (version 14.0; SPSS, Chicago, IL, USA).

### Research Ethics

This project was approved by the Regional Committee for Medical Research Ethics, Eastern Norway. The Norwegian Social Science Data Service has approved the collection and analysis of data.

## Results

### RNA quality control

mRNAs of 16 potential reference genes were quantified by qRT-PCR using equal amounts of RNA templates from every tissue samples. The integrity of RNA (RIN) was ranged from 7.2 to 10.0, with a mean value of 8.9, which indicates good preservation of the RNA; thus suitable for RNA studies.

### Range of expression of candidate endogenous control genes

Table 2 presents the mean Ct and SD values obtained across the candidate endogenous control genes in normal and tumour tissue separated into the two groups of patients; non-metastatic and metastatic colon cancer patients. Additional calculations across all patients are shown in Table 3. Mean Ct values ranged from 8.71 (± 1.31 SD) (18S) across all samples to 26.70 (± 1.69 SD) (TBP). The gene with the lowest standard deviation across all samples was IPO8 which showed an overall SD of 1.28, while the gene with the highest standard deviation across the samples was PGK1 with an overall SD of 2.49. The reference genes displayed a relatively broad range of expression. PGK1 had the widest range of Ct values between 8.35 and 29.83 (mean 21.03 ± 2.49 SD, range of 21.47), while B2M had the narrowest range of Ct values between 15.25 and 23.59 (mean 17.10 ± 1.31 SD, range of 8.34). During the subsequent analyses using Statminer Ct values above 36 are excluded and imputed, because Ct values above this level are not reliable. This quality control will thus influence the Ct ranges.

**Table 2:** Cycle threshold (Ct) values of candidate reference genes divided in the four tissue groups.

Gene symbol	Non-metastatic colon cancer						Metastatic colon cancer					
	
	Tumour			Normal			Tumour			Normal		
	
	Mean	SD	N	Mean	SD	N	Mean	SD	N	Mean	SD	N
18S	8,095	0,546	18	8,440	1,066	18	8,800	1.066	20	9,408	2,035	20
ACTB	20,003	0,765	18	19,949	1,209	18	20,363	1.209	20	20,578	2,673	20
B2M	17,050	0,996	18	17,041	1,002	18	17,217	1.002	20	17,085	1,632	20
GAPDH	18,503	0,722	18	19,502	1,044	18	19,211	1.044	20	20,145	2,541	20
GUSB	23,274	0,375	18	24,081	0,865	18	23,564	0.865	20	24,060	1,981	20
HMBS	25,328	0,736	18	26,577	0,974	18	25,963	0.974	20	27,030	2,436	20
HPRT1	22,795	0,814	18	24,183	0,750	18	23,320	0.750	20	24,264	1,849	20
IPO8	24,575	0,469	18	25,084	0,780	18	25,099	0.780	20	25,529	2,108	20
PGK1	20,322	1,054	18	21,151	1,012	18	20,996	1.011	20	21,573	3,257	20
POLR2A	24,007	0,634	18	24,508	1,061	18	24,933	1.061	20	25,330	2,590	20
PPIA	17,081	0,485	18	18,241	0,906	18	17,506	0.906	20	18,335	1,724	20
RPLP0	19,706	0,637	18	20,647	0,952	18	20,319	0.952	20	21,081	2,002	20
TBP	26,157	0,577	18	26,860	1,035	18	26,649	1.035	20	27,110	2,797	20
TFRC	21,774	0,926	18	23,334	1,030	18	22,679	1.030	20	23,663	2,303	20
UBC	21,285	0,675	18	21,771	1,046	18	21,532	1.046	20	22,044	2,180	20
YWHAZ	23,933	0,723	18	25,041	1,275	18	24,457	1.275	20	25,401	2,174	20

**Table 3:** Cycle threshold (Ct) values of candidate endogenous control genes across all tissue samples.

Gene	Mean	± s.e.m	Standard deviation (SD)	Ct min	Ct max	Ct Range	CtCV%
18S	8.708	0.151	1.314	6.858	16.932	10.073	14,99
ACTB	20.236	0.187	1.630	17.979	31.018	13.039	8,10
B2M	17.101	0.150	1.306	15.251	23.587	8.336	7,69
GAPDH	19.358	0.191	1.661	17.382	30.403	17.382	8,63
GUSB	23.748	0.150	1.309	21.719	32.338	10.619	5,55
HMBS	26.239	0.186	1.625	24.315	36.823	12.508	6,23
HPRT1	23.649	0.164	1.429	21.852	31.617	9.765	6,04
IPO8	25.085	0.146	1.276	23.749	33.903	10.154	5,12
PGK1	21.025	0.285	2.487	8.354	29.829	21.474	10,35
POLR2A	24.717	0.184	1.606	20.827	34.874	14.047	6,54
PPIA	17.978	0.150	1.305	15.980	25.150	9.170	7,34
RPLP0	20.452	0.162	1.413	18.682	29.171	10.489	6,93
TBP	26.704	0.194	1.692	24.858	38.656	13.799	6,38
TFRC	22.878	0.196	1.711	19.907	32.261	12.354	7,53
UBC	21.665	0.163	1.422	19.475	30.387	10.912	6,60
YWHAZ	24.720	0.193	1.685	22.733	32.853	10.120	6,86

*Note*. S.e.m, standard error of mean; CtCV%, Coefficients of variations of candidate reference genes.

### Results of validation programs

**Figure 1:**
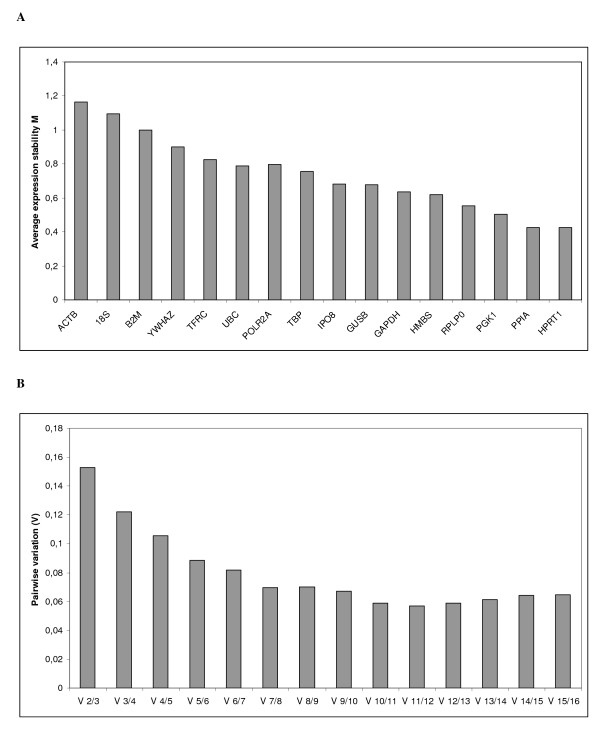
**GeNorm analysis of the candidate reference genes**. (A) Average expression stability values of reference genes. Genes are presented in an increasing order of stability from left to right with ACTB being the least stable gene and HPRT1 and PPIA the most stable genes. (B) Determination of optimal number of control genes for normalization. Every bar represents change in normalization accuracy when stepwise adding more reference genes according to ranking in Figure 1. A. To meet the recommended cut-off value of 0.15 two pr three genes would be satisfactory for normalization.

**Figure 2:**
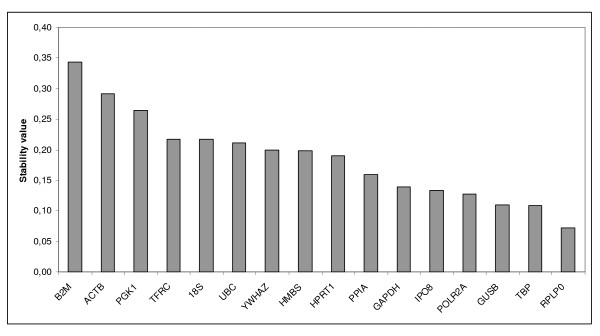
**NormFinder analysis of the candidate reference genes**. Genes are presented in an increasing order of stability from left to right with B2M as the least stable gene and RPLP0 as the most stable gene.

In order to determine the stability of genes and thus find the best endogenous controls, the data were analysed by geNorm and NormFinder. In these analyses, medians were used to replace missing values because they occurred due to inconsistencies between replicates rather than from low expression. The ranking of the gene expression stability values (M) of the tested endogenous control genes using geNorm is illustrated in Figure 1.A. The genes with the highest M, i.e. the least stable genes, gets stepwise excluded until the most stable genes remain. The best two genes are ranked without distinguishing between them. HPRT1 and PPIA were identified as the most stable pair of genes, followed by PGK1 as the third most stable gene. Furthermore, pairwise variation were also calculated using geNorm in order to determine the optimal number of genes required for normalization, Figure 1.B. The analysis showed that HPRT1 and PPIA may be sufficient for calculation of the normalization factor and normalization to genes of interest, since the V2/3 value is in this analysis equal to the cut-off value of 0.15 [19]. However, there is a gradual decrease in the pairwise variability plot and thereby an improvement to the normalization factor by adding additional genes to the calculation. Nevertheless, two or three genes would be satisfactory for normalization according to the cut-off value of 0.15. While geNorm uses a pairwise comparison approach, NormFinder first estimates the intra-group and then the inter-group variability of expression of a control gene [17]. In contrast to the geNorm results, NormFinder ranked RPLP0 as the most stable gene, with TBP and GUSB closely behind as second and third, respectively (Figure 2). However, using this algorithm the combination of IPO8 and PPIA turned out to have a lower stability score than the most stable single gene. Thus this combination is more suitable for normalizing qPCR. There was considerably closer agreement between the geNorm and Normfinder results on the least stable genes, with the order of 4 out of 5 worst ranking genes being identical; ACTB, 18S, B2M and TFRC. These genes had a stability value more than twice so high (geNorm) and more than 3 times so high (NormFinder) as the best ranking genes.

Due to different ranking by geNorm and NormFinder of the most stable genes, cycle threshold coefficient of variation (CtCV%) was calculated for each of them. This calculation was recommended by Caradec et al., 2010, in order to validate the NormFinder and geNorm results [12]. According to the CtCV% calculation, one of the NormFinder pairing genes, IPO8, was ranked as the most stable gene with a CtCV% of 5.12%, which supports the NormFinder result. GUSB (5.5%) and HPRT1 (6.04%) are ranked as the second and third respectively, which do not give identical ranking of results obtain using geNorm and NormFinder. The least stable gene using CtCV% was 18S (14.99%), which was according to geNorm and NormFinder ranked as the second and fifth least stable gene, respectively. The summary of the best ranking genes as determined by each of these calculations is illustrated in Table 4.

**Table 4:** Ranking and best combination of reference genes determined by geNorm, NormFinder and CtCV%.

Rank	GeNorm	NormFinder	CtCV%
1	HPRT1	RPLP0	IPO8 (5.12)
2	PPIA	TBP	GUSB (5.55)
3	PGK1	GUSB	HPRT1 (6.04)
4	RPLP0	POLR2A	HMBS (6.23)
5	HMBS	IPO8	TBP (6.38)
6	GAPDH	GAPDH	POLR2A (6.54)
7	GUSB	PPIA	UBC (6.60)
8	IPO8	HPRT1	YWHAZ (6.86)

Best gene/combination	HPRT1/PP1A	IPO8/PPIA	IPO8

## Discussion

qRT-PCR has been a breakthrough for the quantification of gene expression in many biological systems. In this study we assume that no single gene stays unaffected under malignant development in colon cancer and therefore identify genes with least variation. We identified two pairs of genes, HPRT1/PPIA and IPO8/PPIA, which may be suitable to normalize gene expression data in studies conducted in metastatic and non-metastatic colon cancer patients. In addition, we found that B2M, ACTB and 18S were unstable in the tissue examined. We propose a standardized approach of finding the most suitable reference gene(s) in every qRT-PCR experiment using TLDA.

Complex signalling pathways have been related to the metastatic progression of colon cancer, hence pathway based gene expression assays, often revealed by qRT-PCR, are significant in cancer biology. Publications dealing with colon cancer have reported gene expression studies in metastatic diseases [34,35]. However, the stability of the reference gene expression in metastatic and non-metastatic primary tumours remains crucial. Ramaswamy et al., 2003, described a gene expression signature that distinguished primary and metastatic adenocarcinomas, indicating that the metastatic potential of human tumours is encoded in the bulk of the primary tumour [36], fully in accordance with modern clonal stem cell theories [37]. Hence, one may presume that the metastatic capacity of the primary tumour will influence commonly chosen reference genes.

The most recent study of reference genes in colon cancer was reported by Kheirelseid et al., 2010, where 64 colorectal tumours and tumour associated normal specimens were examined using qRT-PCR followed by three different statistical algorithms, geNorm, NormFinder and qBaseplus [30]. Kheirelseid et al., 2010, found that the combination of two reference genes, B2M and PPIA, more accurately normalized qRT-PCR data in colorectal cancer. This is in concordance with our findings, where PPIA was one of the two genes identified as the most stable pair. In contrast, B2M was identified as one of the most variable genes in the tissue examined. This disparity may be explained by the difference in patient material since Kheirelseid et al., 2010, included all stages of colon cancer and even included rectum tumour samples. Furthermore the percentage of tumour cells in the samples was not addressed. In the study of Kheirelseid et al., 2010, all three algorithms confirmed the selection of the B2M and PPIA pairing as the best combination of reference genes. In the present study however, the geNorm algorithm differs from the results obtained by NormFinder. According to geNorm HPRT1 and PPIA were the most suitable genes for normalization, but NormFinder suggested IPO8 and PPIA. This discrepancy confirms previous results reported by Caradec et al., 2010, concluding that the evaluation of suitable reference genes dramatically differs according to the statistical method used [12]. Caradec et al., 2010, investigated reference genes in four cell lines treated with four different oxygen concentrations, and observed large variations in gene expression results depending of statistical method used. Thus Caradec et al., 2010, recommended Ct coefficients of variation (CtCV%) calculation for each reference gene for validation of the statistical methods. It is defined as the ratio of the standard deviation to the mean. Genes with low CtCV% value indicate more stable expression of those genes. In the present study, IPO8 was the most stable gene on the basis of CtCV% (5.12%), followed by GUSB (5.55%) and HPRT1 (6.04%) as the second and third most stable gene. Using NormFinder IPO8 was one of the genes which were identified as the most stable pair of genes, which may indicate that the CtCV% verifies the NormFinder results. Nevertheless, PPIA, which was suggested by both geNorm and NormFinder as one of the stable pair of genes, was ranked as the tenth most stable gene with a CtCV% of 7.34%. This may be explained by the low Ct mean of this particular gene (18.0), resulting in a relatively high CtCV% despite a low standard deviation. Another aspect which strengthens the results achieved by NormFinder compared with geNorm is the argument that geNorm lacks robustness compared with NormFinder [32]. Reports show that exclusion of one sample in a specific tissue collection led geNorm to change a suggested reference gene (18S) from being an unstable gene to one of the top ranking stable genes [32]. NormFinder also enables estimation of the variation between sample subgroups, like tumour and normal tissue, thus this algorithm can account for heterogeneity in the tested samples, which may be important considering the heterogeneity of the samples studied.

The optimal normalization will vary with study design. The most suitable reference gene in one medical condition may be regulated in other tissues or diseases. Blanquicett et al., 2002, found that 18S, S9 and GUS were the least regulated genes among 15 putative reference genes when examining tumour and normal colorectal and liver tissues [28]. Furthermore, Dydensborg et al., 2006, identified B2M as the most appropriate gene for normalizing colon carcinomas comparing to normal mucosa when they investigated seven colon adenocarcinomas containing both epithelial and stromal cells [29]. B2M was in this study identified as the least stable gene using NormFinder, and the third most variable gene using geNorm. In the present study where the tumour tissue samples consisted of more than 70% tumour cells some of the stromal cells are excluded. This might explain the discrepancies in the ranking of B2M since tumour tissue is heterogeneous and the fraction of different cells may influence the gene expression results. Moreover, different patient groups, including age and clinical background, may also give dissimilarities across studies. Experimental variations may also influence the gene expression results, though using triplicates in the qRT-PCR analysis as used in this study will diminish this variation.

Single assays qRT-PCR are time- and labour-intensive, and require relatively large amounts of cDNA and PCR reagents in multivariate gene expression studies. TLDA overcome these drawbacks since this technique allows for simultaneously detection of expression of up to 384 genes and requires less template cDNA and PCR reagents than routine qRT-PCR [1,31,38-40].

## Conclusions

In this study we applied TaqMan Low Density Array in order to identify reference genes in metastatic and non-metastatic colon cancer. The genes often used for normalization of gene expression data may be unstable and thus not suited for use, and therefore identifying stable reference genes in the specific experiment is vital for the results. The approach described herein can serve as a template to identify valid reference genes in any disease state. However, the optimal statistical approach to identify the best reference gene(s) remains to be determined. In the present study NormFinder and geNorm identified two different pairs of the most stable genes. The use of CTCV% might be a good validation of the two results. Nevertheless, the expression of target genes should be evaluated and a comparison of the effect of each pair of reference genes should be determined.

## Competing interests

The authors declare that they have no competing interests.

## Authors' contributions

LAAS has carried out the molecular biological work, the statistical analyses and drafted the manuscript. SNA has carried out the collection of patients and tissue specimens and has evaluated the percentage tumour cells in the tumour samples, and additionally helped to draft the manuscript. IRKB and MBJ participated in study design and coordination and helped to draft the manuscript. All authors read and approved the final manuscript.
